# Anti-IL6 Autoantibodies in an Infant With CRP-Less Septic Shock

**DOI:** 10.3389/fimmu.2019.02629

**Published:** 2019-11-08

**Authors:** Marketa Bloomfield, Zuzana Parackova, Tamara Cabelova, Iva Pospisilova, Pavel Kabicek, Hana Houstkova, Anna Sediva

**Affiliations:** ^1^Department of Immunology, 2nd Faculty of Medicine, Charles University and Motol University Hospital, Prague, Czechia; ^2^Department of Pediatrics, 1st Faculty of Medicine, Thomayer's Hospital and Charles University, Prague, Czechia; ^3^Department of Clinical Chemistry, 1st Faculty of Medicine, Thomayer's Hospital and Charles University, Prague, Czechia

**Keywords:** interleukin 6, C-reactive protein, anti-IL6 autoantibodies, tocilizumab, siltuximab, sarilumab

## Abstract

**Background:** Interleukin-6 (IL-6) is a pleiotropic cytokine with a multitude of pro-inflammatory effects. Serum C-reactive protein (CRP) is an acute phase protein induced mainly by IL-6 in response to inflammatory conditions, particularly infection. The biological functions of CRP include opsonisation, induction of phagocytosis, complement activation, or chemotaxis enhancement. Factors interfering with IL-6-mediated recruitment of innate immune responses, such as the presence of anti-IL6 antibodies, may therefore compromise the host resistance to microbial pathogens. This has major implications for the use of IL-6-targeting biologics, such as tocilizumab or sarilumab in rheumatologic, immune dysregulation diseases, and cancer.

**Case presentation:** 20-month-old Czech female developed severe septic shock with clinical and laboratory signs of systemic inflammation but no increase of CRP or IL-6. The offending pathogen was most likely *Staphylococcus aureus*, detected in a throat swab; the response to antibiotic treatment was prompt. A defect in the integrity of IL-6/CRP axis was suspected and verified by the detection of neutralizing IL-6 antibodies in the serum of the child.

**Conclusion:** We report a first case of systemic bacterial infection in a patient with anti-IL6 autoantibodies. Disturbed IL-6 signaling, whether iatrogenic by targeted IL-6 blockade or endogenous due to the presence of autoantibodies against IL-6, represents a risk factor for increased infectious susceptibility. Patients with severe bacterial infection without elevation of CRP should be examined for the presence of anti-IL6 autoantibodies.

## Background

Interleukin 6 (IL-6), originally described as B-cell stimulatory factor in 1985, is now known as a pleiotropic cytokine with multitude of key biological functions, including inflammatory and immune responses, hematopoiesis, and oncogenesis. It is transiently produced by immune cells, such as monocytes and macrophages, but also by other cell lineages upon various stimuli, e.g. infection or tissue injury (1). IL-6 binds to its receptor, which exists in two forms; a membrane-bound protein or a soluble form. In short, upon IL-6 binding the downstream signaling molecules Janus kinases (JAKs) recruit either signal transducer and activator of transcription 3 (STAT3) or mitogen-activated protein kinases (MAPKs) via receptor-associated molecule gp130. This initiates the transcription of IL-6-inducible genes (2) and results, inter alia, in the production of proteins such as C-reactive protein (CRP), fibrinogen and serum amyloid A.

CRP is an acute phase reactant produced by hepatocyte-derived IL-6-dependent biosynthesis in inflammatory conditions, particularly in response to infection. Its biologic functions are promotion of innate immune processes, including opsonisation, complement activation, induction of release of pro-inflammatory cytokines or promotion of phagocytosis and chemotaxis (3). CRP serum levels begin to rise by 6 hours and peak within 2–3 days from induction (4).

Procalcitonin (PCT) is produced in health in thyroid cells and immediately converted to the hormone calcitonin. On the other hand, the inflammatory PCT is released mainly by adipocytes and white blood cells, triggered by various microbial peptides or inflammatory mediators such as IL-6 or tumor necrosis factor-alpha (TNFα). PCT is utilized as a diagnostically accurate tool for bacterial infection and a useful discriminator of sepsis. Its levels increase more rapidly than CRP, between 2 and 6 hours and peak within 6–24 hours during infection (5).

Sepsis is defined as systemic inflammatory response to infection (6) or, more recently, as life-threatening organ dysfunction caused by a dysregulated host response to infection (7). Septic shock is clinically identified as sepsis with cardiovascular dysfunction (6). During sepsis, an array of cytokines and chemokines is produced, such as interleukin 1β (IL-1β), IL-6, TNF-α, or soluble CD14 (5). Currently, IL-6, CRP and PCT are the most commonly used biomarkers of sepsis, which severity and outcome prediction capacity is of high clinical research interest.

Given the role of IL-6 in immune responses, an enhanced infectious susceptibility is a rational concern in any therapeutic strategy targeting IL-6 signaling, e.g. IL-6 receptor (IL6R) (tocilizumab, sarilumab) or IL-6 (siltuximab), increasingly utilized in treatment of rheumatoid arthritis (RA), juvenile idiopathic arthritis (JIA), or Castleman's disease (8).

To date, three patients only were reported to suffer severe bacterial infections while having detectable neutralizing antibodies to IL-6 and impaired acute phase response (9, 10). The hereby-presented case describes the fourth such patient, who is also the first to present with severe systemic inflammatory response.

## Case Presentation

### Clinical Vignette

A Czech female was born in 36th gestational week to a mother with history of intravenous methylamphetamine abuse during pregnancy. She suffered severe perinatal asphyxia and multiple ileal perforations requiring a stoma, which was closed at 4 months. At the age of 5 months, she suffered another ileal perforation, during which an increase of CRP (86,7 mg/L) and leucocytosis (19,7 × 10^*^9/L) were noted. The subsequent infectious susceptibility was inconspicuous; she thrived relatively well, received hexavalent combined vaccine (diphtheria, tetanus, pertussis, poliomyelitis, *Haemophilus influenzae type B*, and hepatitis B) and developed within the neurologic limitations of her perinatal insult. At the age of 20 months, she suffered a short paroxysm of generalized seizures in a second day of fevers of 38, 0–38, 5°C. Upon admission, she presented with dehydration, circulatory instability with hypotension, tachycardia, tachypnea, and anuria. Her laboratory workup showed mild leukopenia 5.8 × 10^*^9/L (ref. range 6.0–17.5), thrombocytopenia 64 × 10^*^9/L (ref. range 150–450), severe electrolyte imbalance, increased renal parameters (creatinin 132 umol/L, ref. range 8–45; urea 25 mmol/L, ref. range 3.2–9.0), signs of rhabdomyolysis (increased aspartate aminotransferase, serum creatine kinase, myoglobin) and elevated D-dimers, activated partial thromboplastin time but unincreased fibrinogen 2.82 g/L (ref. range 1.45–3.48). An extreme elevation of PCT 378.0 ug/L (electrochemiluminescence, ref. range 0.0–0.5) but, curiously, no increase of CRP 2.9 mg/L (immunoturbidimetry, ref. range 0.0–5.0) or IL-6 16.2 ng/L (electrochemiluminescence, ref. range 0.0–20.0) were noted. *Staphylococcus aureus* was cultured from throat swabs, other microbiologic investigations were negative, including blood cultures. She was diagnosed with septic shock, required massive intravenous volume expansion and received 10 days of antibiotic treatment (third generation cephalosporin and gentamicin) that controlled the infection and the laboratory parameters normalized. During the following 6 months, she experienced no other infections.

**Figure 1:**
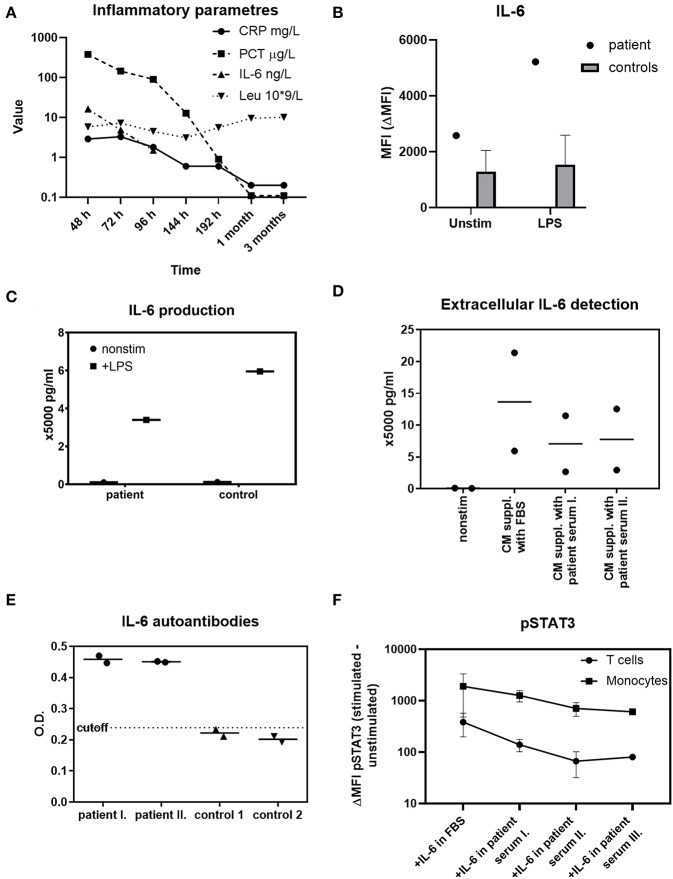
Investigations of IL-6 functions in the patient. **(A)** The dynamics of IL-6, CRP, PCT, and absolute leukocytes count in various time-points during sepsis, 1 and 3 months afterwards (h-hours from onset of fever, m-month/s). An extreme elevation of PCT is not accompanied with IL-6 or CRP increases over the normal reference value. Reference ranges: CRP 0.0–5.0 mg/L; PCT 0.0–0.5 μg/L; IL-6 0.0–20.0 ng/L; Leu 6.0–17.5 × 10^9^/L. **(B)** Intracellular IL-6 production in patient's monocytes at the time of sepsis compared to 59 healthy controls' monocytes after 1 μg/ml LPS determined with flow cytometry. Unstimulated state is expressed as MFI (mean fluorescence intensity). The effect of LPS stimulation is expressed as ΔMFI (stimulated minus unstimulated MFI). The synthesis of IL-6 by patient's monocytes is unskewed. **(C)** Detection of extracellular IL-6 from patient's PBMCs after 1 μg/ml LPS stimulation determined by ELISA. The release of IL-6 into extracellular space by patient's cells is normal. **(D)** Suppression of extracellular IL-6 by patient's serum analyzed by ELISA. Healthy age-matched controls' PBMCs (*n* = 2) were stimulated with LPS, cultivated in complete media (CM) supplemented either with fetal bovine serum (FBS) or 10% patient serum in time of sepsis (I) and 1 month later (II) The amount of IL-6 detected in the presence of patient's serum is decreased in both time-points. **(E)** Anti-IL-6 autoantibodies detection in patient serum obtained in time of sepsis (I), 1 month later (II), and in 2 healthy age-matched controls with ELISA. The patient's serum, but not the control serum, contains anti-IL6 autoantibodies. OD, optical density. **(F)** STAT3 phosphorylation (pSTAT3) in control (*n* = 2) T cells and monocytes after 10 ng/ml recombinant IL-6 stimulation. The peripheral blood was stimulated with IL-6 diluted in PBS containing 20% patient serum obtained in sepsis (I), 1 month later (II), 3 months later (III), or in fetal bovine serum (FBS). The patient's serum decreases the pSTAT3 signal in all three time-points. Data are expressed as ΔMFI (stimulated minus unstimulated MFI). MFI, mean fluorescence intensity.

The patient's basic immune profiling suggested no gross abnormality (Table 1). However, intrigued by the peculiar dynamics of the inflammatory markers during sepsis, especially the lack of IL-6 and CRP response along the high PCT elevation (Figure 1A), we prompted investigation of the integrity of IL-6 signaling axis, which we tested in the following steps.

**Table 1:** Patient's basic immune profile.

**Patient's immune profile**	**Value**	**Age-matched reference values**
Leukocytes (cells/μL)	10,100	6,000–17,000
Lymphocytes (cells/μL)	7,180	2,900–12,400
Neutrophils (cells/μL)	1,550	1,300–8,200
Monocytes (cells/μL)	1,010	150–1,280
Eosinophils (cells/μL)	610	0–1,200
CD3^+^ (%^a^, cells/μL)	76 ↑/ 5,457 ↑	56–75/1,400–3,700
CD3^+^ CD4^+^ (%^a^, cells/μL)	42/3,016 ↑	28–47/700–2,200
CD3^+^ CD8^+^ (%^a^, cells/μL)	27/1,939 ↑	16–30/490–1,300
Naïve CD4^+^ (%^b^)	36	36–97
(CD3^+^CD4^+^CD45RA^+^CD27^+^)		
Naïve CD8^+^ (%^c^)	19	19–95
(CD3^+^CD8^+^CD45RA^+^CD27^+^)		
CD19^+^ (%^a^, cells/μL)	18/1,292	14–33/390–1,400
Naïve CD19^+^ (%^d^)	91	49–100
(CD19^+^CD27^−^IgD^+^)		
Switched memory CD19^+^(%^d^)	3↓	5–25.6
(CD19^+^CD27^+^IgD^−^)		
CD16^+^/CD56^+^ (%^a^, cells/μL)	4.4 /316	4–17/130–720
Immunoglobulins		
IgG (g/L)	4.76↓	5.53–10.20
IgG1(g/L)	2.4 ↓	2.90–8.50
IgG2 (g/L)	1.33	0.45–2.60
IgG3 (g/L)	0.42	0.15–1.13
IgG4 (g/L)	0.61	0.01–0.79
IgA (g/L)	0.31↓	0.33–0.91
IgM (g/L)	0.47	0.47–1.55
IgE (IU/mL)	60.6↑	0.0–30.0
IgD (IU/mL)	<5.65	0.0–100.0
a-tetanus, a-diphtheria, a-hemophilus postvaccination IgG	Normal	NA
Autoantibodies (ANA, ENA, a-dsDNA, RF, ANCA, a-TPO, a-TG, a-TSHR, a-EM, a-TTG, a-GD)	Neg	NA
Complement activation (%):		Reference ranges
Classic pathway	94	69–129
Alternative pathway	53.4	30–113
MBL pathway	0.4↓	>10
Burst test PMA and *E.coli*, NBT test	Normal	NA

a *% of total peripheral lymphocytes*.

b *% of CD4^+^*.

c *% of CD8^+^*.

d *% of CD19^+^*.

*NA, not applicable; SCIG, subcutaneous immunoglobulins; Neg, negative; ANA, antinuclear antibodies; ENA, extractable nuclear antigen antibodies; a-dsDNA, anti-double stranded DNA antibodies; RF, rheumatoid factor IgG, IgA and IgM; ANCA, anti-neutrophil cytoplasm antibodies; a-TPO, anti-thyreoperoxidase antibodies; a-TG, anti-thyreoglobulin antibodies; a-TSHR, TSH receptor antibodies; a-EM, anti-endomisium IgG and IgA antibodies; a-TTG, anti-tissue transglutaminase antibodies IgG and IgA; a-GD, anti-deamidated gliadin IgA and IgG; MBL, mannan binding lectine; PMA, Phorbolmyristate acetate; NBT, nitroblue tetrazolium. ↑,↓, value above, below reference range, respectively*.

### The Ability to Synthetize IL-6 by Patient's CD14^+^ Monocytes Is Normal

In order to establish a normal cellular ability to produce IL-6, patient's whole blood was stimulated with lipopolysaccharide (LPS) in presence of Brefeldin A. Flow cytometric trace of IL-6 (Figure 1B), IL-1β and TNFα (Supplementary Figure 1) in CD14^+^ monocytes was analyzed. We observed an increased unstimulated production of IL-6 and IL-1β in the time of sepsis, which further increased after LPS stimulation, demonstrating an unskewed ability to synthetize these cytokines. The production of TNFα was similar to healthy controls.

### The Ability to Release IL-6 Into Extracellular Space by Patient's Cells Is Normal

Having established a normal intracellular IL-6 synthesis, we sought to determine the patient's cells ability to release the cytokine extracellularly. Patient's peripheral blood mononuclear cells (PBMCs) were stimulated with LPS overnight. The supernatants were harvested and the IL-6 was determined using a commercial IL-6 Elisa assay. We found the PBMCs of the patient to be capable of substantial IL-6 extracellular release, even if slightly decreased compared to a healthy control (Figure 1C).

### The Patient's Serum Has IL-6 Neutralizing Property

Because of the patient's uncompromised ability to produce CRP at the age of 5 months, we hypothesized that an induction of anti-IL6 antibodies (abs) may underlie the acquired IL-6/CRP irresponsiveness. The healthy donors' PBMCs were stimulated according to the protocol above or left unstimulated in complete media (CM) supplemented either with patient's serum obtained from 2 different time points (in time of sepsis and 1 month later) or with fetal bovine serum (FBS). We noted a profound decrease of the cytokine in the presence of patient's serum. This indicated that the patient's serum contained a component interfering with the IL-6 detection (Figure 1D).

### The Patient's Serum Contains Anti-IL6 Autoantibodies

The anti-IL6 abs were detected in the patient's and healthy donors' sera using a commercial Elisa kit (MyBiosource, details available in List of Methods). While the control samples were negative for anti-IL6 abs, the patient's serum was found positive in time of sepsis as well as 1 month after the infection (Figure 1E).

### The Patient's Serum Decreases the Intracellular IL-6-Mediated Signal Transduction

Finally, we investigated whether the IL-6 abs found in the patient's serum cause a corresponding depression of IL-6 signal transduction downstream of IL-6 receptor (IL6R). To do this, we cultivated recombinant IL-6 in phosphate-buffered saline (PBS) supplemented with patient's serum obtained from 3 different time-points (in sepsis, 1 and 3 months later) or with FBS. Then, control full blood (*n* = 2) was stimulated with IL-6 in the respective media and STAT3 phosphorylation (pSTAT3) was analyzed in peripheral T cells and monocytes. We observed a significantly lower pSTAT3 signal from samples containing patient's serum from all 3 time-points (Figure 1F). The results suggest that the IL-6 abs have a neutralizing effect and indicate the persistence of the IL-6 abs even beyond acute phase of the infection.

## Discussion and Conclusions

We present a child with septic shock, which most likely developed on the grounds of serum anti-IL6 autoAbs. Based on the lack of detectable IL-6, CRP and fibrinogen response during a clinically manifested systemic inflammation, together with the disturbed IL-6/STAT3-mediated signaling observed in cells exposed to patient's serum, we suggest that these abs have neutralizing property and contributed to the severity of the infection. Meanwhile, the undisturbed functionality of other proinflammatory cytokines, such as IL-1β or TNFα probably explains the patient's retained ability to develop other features of inflammatory response, such as fever or increased PCT. PCT, a strong IL-6 independent biomarker of bacterial infection, rises sooner than CRP. Yet, due to its biologic half-life its increase should later overlap with CRP elevation. Therefore, the PCT/IL-6/CRP discrepancy during the acute phase of infection in our patient supports the hypothesis of isolated defect in IL-6-mediated CRP induction.

To our knowledge, this is the first patient with anti-IL6 autoAbs reported to suffer a severe systemic infection. Previously, Puel et al. reported a Haitian boy with recurrent *Staphylococcus aureus* subcutaneous abscesses and cellulitis (9) and Nanki et al. referred two adult Japanese patients presenting with *Staphylococcal aureus cellulitis* and *Streptococcus intermedius* and *Escherichia coli empyema* (10) (Supplementary Table 1).

Eventhough very likely, a causative link between the anti-IL6 abs and the infectious susceptibility may not be unequivocally established in our patient. With no human IL-6 deficiency reported to date, the corresponding phenotype and the exact underlying molecular mechanisms of such defects are yet to be elucidated. Nevertheless, some clues may be derived from patients with genetic loss of proteins involved in IL-6/gp130/STAT3 signaling pathway. Two patients were recently reported to harbor homozygous IL6R mutations resulting in a phenotype of recurrent infections, absence of CRP increase during acute phase of clinically apparent infections, elevated IgE and eczema (11). A single case of bilallelic gp130 mutation has been described to present as early onset severe bacterial infections including *Staphylococcus aureus*, eczema, impaired acute phase response and increased IgE (12). Similar features are associated with hypomorphic STAT3 mutations, which constitute the autosomal dominant HyperIgE syndrome (AD HIES) [reviewed in (13)]. Additionally, an IL-6 knockout murine model was shown to develop normally, but the inflammatory acute-phase response after tissue damage or infection was severely compromised (14).

The apparently impeded resistance to *Staphylococcus aureus* in subjects with IL-6 signaling disruption is intriguing. Various primary immunodeficiencies (PIDs) predispose to abnormal, but not selective staphylococcal susceptibility, such as X-linked chronic granulomatous disease, NEMO deficiency syndrome, IRAK-4 deficiency, MyD88 deficiency, or DOCK8 deficiency. Mechanistically, the most relevant to our case is the STAT3 loss-of-function AD HIES, classically hallmarked by recurrent staphylococcal skin and lung infections, and the IL6R deficiency with both reported cases suffering with staphylococcal infections (11). Such similarity strongly suggests that the functional integrity of IL-6/STAT3 pathway is particularly important in antistaphylococcal immunity, however, the exact mechanism is not yet clear. It may involve the lack of CRP-mediated protection or other aspects, such as disturbed Th17 functions or diminished circulating T follicular helper cell induction, which was observed in AD HIES and IL6R deficiency.

Interestingly, several PIDs have recently been coupled with their phenocopies that arise from the presence of anti-cytokine abs. For example, an increased susceptibility to weakly virulent mycobacteria due to IFN-γ autoAbs resembles a rare PID called Mendelian susceptibility to mycobacterial diseases due to monogenic defects in IL-12/IFN-γ circuit. Similarly, abs against Th17-related cytokines IL-17A, IL-17F, IL-22, IL-23 underlie increased susceptibility to fungal infections resembling chronic mucocutaneous candidiasis due to various genetic etiologies [reviewed in (15)].

Naturally occurring anti-IL6 autoAbs were found in 0.1% healthy population but these are likely low concentration and lack the neutralizing property due to their low affinity (16). As with the majority of human autoantibodies, the reason why our patient developed blocking anti-IL6 autoAbs is unknown. A genetic predisposition might play a role. However, having been able to develop a normal acute phase response, including a CRP increase, at the age of 5 months, a single gene inborn error is unlikely. Of note, the two adult subjects reported previously to produce blocking IL-6 abs were not affected with increased infectious susceptibility until 56 and 67 years of age (10). These aspects suggest that “multiple hits” may be required in the disease pathophysiology. Also, our patient's severe perinatal history may underlie an immune dysbalance, owing to early abnormal exposure to pathogens or self-antigens, which may result in autoAbs induction. Nevertheless, no other clinically relevant abs were detected in the patient's blood.

Finally, two IL6R and one IL-6 blocking agents are currently approved and widely used in diseases such as RA, JIA and Castleman's disease (8). While some studies identified a higher incidence of severe infections in RA patients receiving anti-IL6R blockade compared to anti-TNFα biologics (17, 18), others did not (19). However, the suppression of CRP in RA patients receiving monoclonal anti-IL6R abs has been well-recognized, in fact CRP has been suggested as an outcome predictor and treatment monitoring tool (20). In the same context, CRP has also been reported to be a poor predictor of severe infectious complications (21). Several ongoing clinical trials investigate the efficacy and tolerability of IL-6 targeting in various other immune dysregulation or oncologic diseases and novel compounds interfering with IL-6 signaling are being rapidly developed (8). Therefore, given the severity of presentation in our patient, we suggest that extra care in exercised in patients receiving IL-6 blocking agents, especially when administered together with other immune suppressant drugs.

Of note, an excess of soluble IL-6R, which would bind the IL-6 and interfere with its detection was not excluded in our patient. This is a limitation of our study, however the documented presence of anti-IL6 autoAbs amply explains the observed phenomena.

To conclude, disturbed IL-6/STAT3/CRP axis due to endogenous production of anti-IL6 autoAbs was a likely cause of severe septic shock in our patient who failed to mount an efficient acute phase response. We suggest that patients with severe bacterial infection without elevation of CRP should be examined for the presence of anti-IL6 antibodies.

## Data Availability Statement

All data generated or analyzed during this study are included in the article/Supplementary Material and are also available from the corresponding author on reasonable request.

## Ethics Statement

Ethical review and approval was not required for the study on human participants in accordance with the local legislation and institutional requirements. Written informed consent to participate in this study and for publication was provided by the participants' legal guardian/next of kin.

## Author Contributions

MB treated the patient, established the hypothesis and wrote the manuscript. ZP designed and performed the experiments and co-wrote the manuscript. TC treated the patient and revised the manuscript. IP performed the routine tests and revised the manuscript. PK and HH supervised the patient treatment, manuscript preparation and revisions. AS supervised the experiments, manuscript preparation and revisions. All authors have contributed in a substantive and intellectual manner.

### Conflict of Interest statement

The authors declare that the research was conducted in the absence of any commercial or financial relationships that could be construed as a potential conflict of interest.

## Supplementary Material

Supplementary Figure 1 Cytokine production by patient's monocytes in time of sepsis: IL-1β and TNFα production determined by flow cytometry compared to 65 and 30 healthy controls, respectively. Unstimulated state is expressed as MFI (mean fluorescence intensity). LPS stimulation is expressed as ΔMFI (stimulated—unstimulated MFI).

Supplementary Table 1 An overview of reported cases of patients with anti-IL-6 autoantibodies and severe bacterial infections.

## Abbreviations

Abs: antibodies
AD HIES: autosomal dominant HyperIgE syndrome
Anti-IL6 abs: antibodies against interleukin 6
Anti-IL6 autoAbs: autoantibodies against interleukin 6
CM: complete medium
CRP: C reactive protein
DOCK8: dedicator of cytokinesis 8
FBS: fetal bovine serum
IFN-γ: interferon gamma
IL12, IL-17A, IL-17F, IL-22, IL-23: interleukin of corresponding enumeration
IL-6: interleukin 6
IL6R: interleukin 6 receptor
IRAK-4: interleukin 1 receptor-associated kinase 4
JAKs: Janus kinases
JIA: juvenile idiopathic arthritis
LPS: lipopolysaccharide
MAPKs: mitogen-activated protein kinases
MyD88: myeloid differentiation primary response 88
NEMO: nuclear factor-kappa B essential modulator
PBMC: peripheral blood mononuclear cells
PBS: phosphate-buffered saline
PCT: procalcitonin
pSTAT3: phosphorylated signal transducer and activator of transcription 3
RA: rheumatoid arthritis
ref.range: reference range
STAT3: signal transducer and activator of transcription 3
TNFα: tumor necrosis factor-alpha.
