# Supplementary figures and images for: Anti-IL6 Autoantibodies in an Infant With CRP-Less Septic Shock
Source: Front Immunol. 2019 Nov 8;10:2629. doi: 10.3389/fimmu.2019.02629 (PMC6857097; doi:10.3389/fimmu.2019.02629)

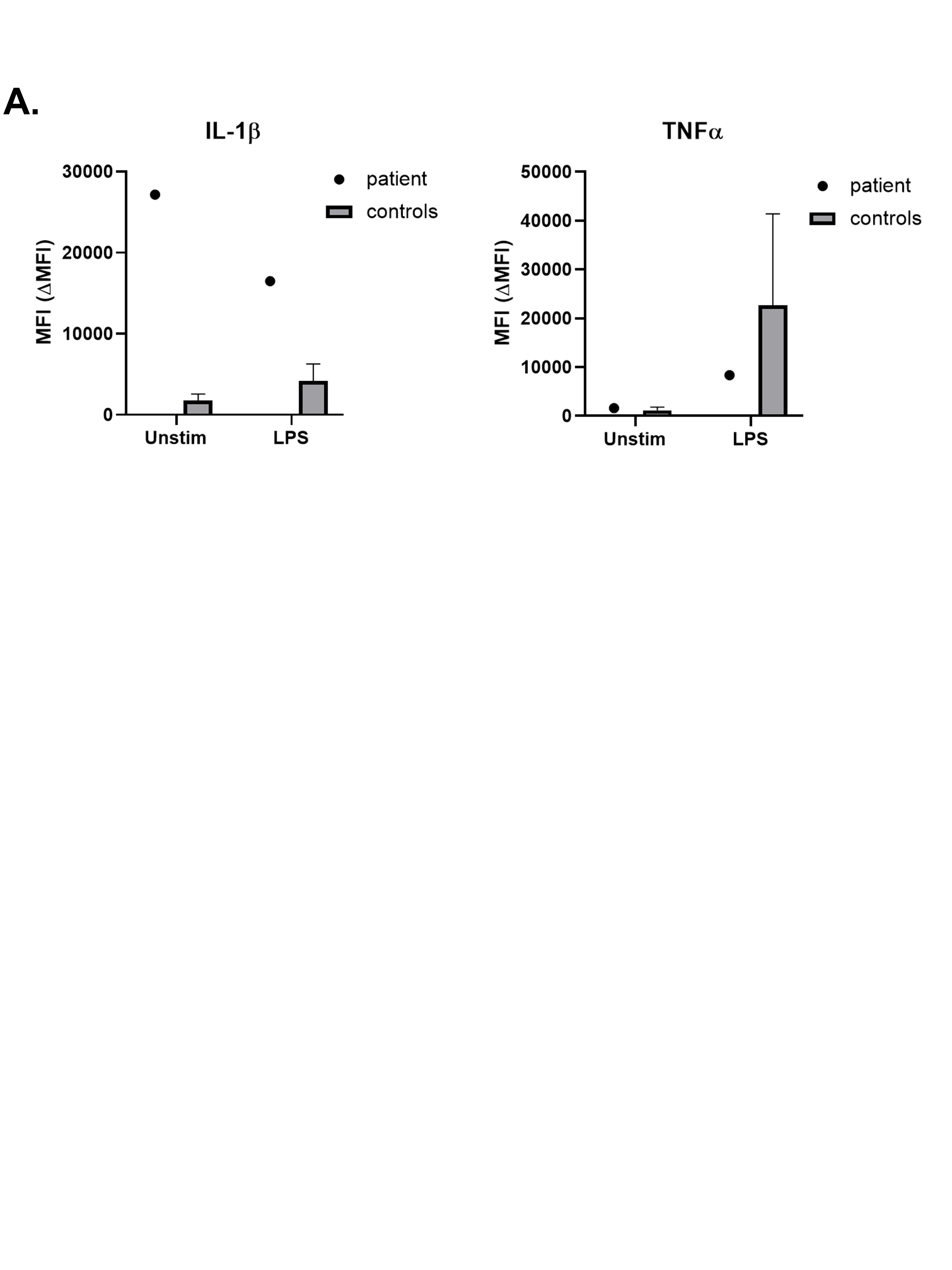

Supplement: Supplementary Figure 1 — Cytokine production by patient's monocytes in time of sepsis: IL-1β and TNFα production determined by flow cytometry compared to 65 and 30 healthy controls, respectively. Unstimulated state is expressed as MFI (mean fluorescence intensity). LPS stimulation is expressed as ΔMFI (stimulated—unstimulated MFI). [file Image_1.TIF]
