# Supplementary material for: Anti-IL6 Autoantibodies in an Infant With CRP-Less Septic Shock
Source: Front Immunol. 2019 Nov 8;10:2629. doi: 10.3389/fimmu.2019.02629 (PMC6857097; doi:10.3389/fimmu.2019.02629)
Supplement: Supplementary Table 1 — An overview of reported cases of patients with anti-IL-6 autoantibodies and severe bacterial infections. [file Table_1.DOCX]

|  | Pt 1  (Nanki et al.) | Pt 2  (Nanki et al.) | Pt 3  (Puel et al.) | Pt 4  (Bloomfield et al.) |
| --- | --- | --- | --- | --- |
| Age at presentation | 67 years | 56 years | 11 and 29 months | 20 months |
| Sex | male | female | male | female |
| Presentation | thoracic empyema | multiple subcutaneous abscesses | recurrent cellulitis and subcutaneous abscesses | sepsis |
| Pathogen | *Escherichia coli, Streptococcus intermedius* | *Staphylococcus aureus* | *Staphylococcus aureus* | likely *Staphylococcus aureus* |
| Body temperature at presentation | 36,6°C | 37,4°C | 39,0°C | 38,5°C |
| Course of the infection | deceased of respiratory failure | recovered | recurrent but recovered | severe septic shock, fully recovered |
| Comorbidities | none reported, no history of severe bacterial infections | rheumathoid arthritis, no history of severe bacterial infections | varicella at 11 months, no history of severe bacterial infections | drug abuse in pregnancy, prematurity, perinatal asphyxia, multiple ileal perforations |
| C-reactive protein | 0,1 mg/L | 0,5 mg/L | <5mg/L | 2,9 mg/L |

Supplementary Table 1: An overview of reported cases of patients with anti-IL-6 autoantibodies and severe bacterial infections
