# Supplementary material for: Anti-IL6 Autoantibodies in an Infant With CRP-Less Septic Shock
Source: Front Immunol. 2019 Nov 8;10:2629. doi: 10.3389/fimmu.2019.02629 (PMC6857097; doi:10.3389/fimmu.2019.02629)
Supplement: Supplementary file 3 [file Data_Sheet_1.docx]

# Supplementary data

# List of methods

## Intracellular IL-6, IL-1β or TNFα production

Intracellular detection of IL-1β was performed in heparinized whole blood stimulated with 100ng/ml LPS (lipopolysaccharide, Sigma Aldrich, St. Luis, USA) 4 h in a presence of Brefeldin A (BioLegend, San Diego, USA). For analysis of IL-6 and TNF-α production, heparinized whole blood was stimulated with 100ng/ml LPS, Brefeldin A was added after 2 h and samples were left for another 4 h in 37°C. Cells were labeled with anti CD14^-^PE-Cy7 (Exbio, Prague, Czech Republic). Following the lysis of RBC using BD Lysing solution (BD Biosciences, San Jose, USA) and fixation/permeabilisation using eBioscience protocol, cells were stained with antibodies against respected cytokines: IL-1β-PE (Thermo Fisher, Waltham, USA), IL-6-APC or TNF-α-BV421 (BioLegend) for 30 min. Samples were washed and measured on Canto II instrument (BD Biosciences). Data was analyzed using FlowJo software (TreeStar, Ashland, OR, USA).

## Extracellular IL-6 detection

**2x10^5^ peripheral blood mononuclear cells (PBMCs) were resuspended in 200μl of RPMI media containg 5% patient serum or in complete media containg 5% commertially available human PHS plasma (Sigma Aldrich) used as positive control. Cells were stimulated** with 1μg/ml of LPS or left unstimulated for 24 hours. **The supernatants were harvested and the IL-6 was determined using IL-6 Elisa according to manufacturers instructions.**

## IL-6 autoantibodies detection

The autoantibodies presence was detected in patient and healthy donors’ sera using commercial available Elisa kit (My BioSource, San Diego, CA, USA): The method is based on anti-IL6 autoantibody antibody-anti-IL6 autoantibody antigen interactions (immunosorbency) and an HRP colorimetric detection system to detect anti-IL6 autoantibody antigen targets in samples. The ELISA detects IgGs. The microtiter plates were pre-coated with the antigen. Samples are pipetted into the wells with anti-human IgG conjugated Horseradish Peroxidase (HRP). Any antibodies specific for the antigen present will bind to the pre-coated antigen. Following a wash to remove any unbound reagent, a substrate solution is added to the wells and color develops in proportion to the amount of human anti-GM1 antibody (IgG) bound in the initial step. The ELISA Kit is designed to detect native, not recombinant, anti-IL6 autoantibody.

## STAT3 phosphorylation

Recombinant (rh) IL-6 was incubated in phosphate buffered saline supplemented with 20% patient serum or fetal bovine serum (FBS) for 2 hours in 4°C in dark. Full blood was stimulated with 10ng/ml “patient serum-derived” rhIL-6, “FBS-derived” rh IL-6 or left untreated for 5 minutes in 37°C. Subsequently, the cells were fixed using 4% formaldehyde for 10 minutes at 25°C, erythrocytes were lysed using 0.1% Triton X-100 (Sigma Aldrich, Darmstadt, Germany) for 15 minutes at 37°C and the leukocytes were permeabilized using 80% ice-cold methanol for 30 minutes. Samples were labelled with antibodies against CD3 – Alexa 700, CD14 – PE-Dy594 (both from Exbio) and intracellular signaling was detected using anti-phosphoSTAT3 (Tyr705) - PE antibody (BD Bioscience). Samples were measured on Aria II instrument (BD Biosciences). Data was analyzed using FlowJo software (TreeStar).
